# Safety of slow‐pulsed transcranial electrical stimulation in acute spike suppression

**DOI:** 10.1002/acn3.50934

**Published:** 2019-11-11

**Authors:** Mark D. Holmes, Rui Feng, Mackenzie V. Wise, Chengxin Ma, Ceon Ramon, Jinsong Wu, Phan Luu, Jidong Hou, Li Pan, Don M. Tucker

**Affiliations:** ^1^ Regional Epilepsy Center Department of Neurology University of Washington Seattle Washington; ^2^ Department of Neurosurgery Huashan Hospital Fudan University Shanghai China; ^3^ Department of Electrical Engineering University of Washington Seattle Washington; ^4^ Brain Electrophysiology Laboratory Company Eugene Oregon; ^5^ SleepIQ Labs San Jose California

## Abstract

We examined the effects of slow‐pulsed transcranial electrical stimulation (TES) in suppressing epileptiform discharges in seven adults with refractory epilepsy. An MRI‐based realistic head model was constructed for each subject and co‐registered with 256‐channel dense EEG (dEEG). Interictal spikes were localized, and TES targeted the cortical source of each subject's principal spike population. Targeted spikes were suppressed in five subject's (29/35 treatment days overall), and nontargeted spikes were suppressed in four subjects. Epileptiform activity did not worsen. This study suggests that this protocol, designed to induce long‐term depression (LTD), is safe and effective in acute suppression of interictal epileptiform discharges.

## Introduction

Because many drug‐resistant epileptic patients are not viable candidates for neurosurgical resection,^1^ alternative treatments are needed to better control seizures. An ideal approach is to develop novel technologies that are safe, noninvasive, and effective in suppressing brain excitability, thereby preventing seizures. Progress has been made in recent years, both in understanding the physiological regulation of cortical excitability^2^ and in controlling excitability with noninvasive electrical and magnetic neuromodulation^3^. Encouraging results have been obtained through inducing long‐term depression (LTD) with transcranial magnetic stimulation^4^ and several studies that employed cathodal transcranial direct current stimulation have shown promise in suppressing seizures 5, 6, 7, 8.

In the present research, we implemented a slow‐pulsed transcranial electrical stimulation (TES) protocol, which induces LTD,^3^, ^4^ in subjects where realistic head models were constructed using each subject's own MRI as basis for the model. Accurate targeting of the cortical focus of epileptic spikes was achieved with current source‐sink patterns derived from the combination of a high‐resolution head conductivity model and a 256‐channel dense EEG (dEEG) system.^9^, ^10^ Compared to conventional 32‐channel EEG, high channel density recordings reduce interelectrode distances, thereby approaching the theoretical “spatial Nyquist,”^11^ and leading to the capability of extracting more useful neural information and reducing spatial localization error rates 12. Integration of the TES delivery system with the dEEG system permits targeting of spike onset zone, through analysis of spike‐generated voltage patterns, with the same electrodes that localize the spike in the first place.

## Subjects and Methods

### Subjects

Subjects 18 years and older (14 years and older in Shanghai) with medically refractory localization‐related epilepsy were invited to participate in this trial for suppressing epileptic discharges with electrical pulses**.** Four men and three women, with mean age of 35 years (range 23–54) completed this study. Regulatory approval and issuance of an investigational device exemption was obtained from the US FDA (IDE G150023; study NCT02516228).^13^ Subject inclusionary and exclusionary criteria are summarized in Table 1. The research protocol and consent forms received approval from the human subjects institutional review boards of the University of Washington and Fudan University. Patients 1–4 were evaluated and treated at the University of Washington Regional Epilepsy Center, Seattle, WA, and patients 5–7 were evaluated and treated at the Epilepsy Unit at Huashan Hospital, Shanghai, China.

**Table 1:** Inclusionary and exclusionary criteria.

Criteria	Description
Inclusion	
1	Harborview Medical Center (Seattle, USA): Between 18 and 60 years old. Huashan Hospital (Shanghai, China): Between 14 and 60 years old.
2	Partial onset seizures (simple or complex) with failure of adequate seizure control after prior use of at least two antiseizure drugs at effective doses.
3	A clearly identified and localizable focus of epileptiform discharges, as defined by the discharges (epileptiform spikes or sharp waves) and as identified by dEEG assessment through one or more routine clinical dEEG evaluations.
4	Two or more partial seizures, with or without secondary generalization, in the last month, but less than 10 seizures per day.
5	Antiseizure drug regimen has remained unchanged for the month before study entry, and there is reasonable likelihood of stability for the duration of the study, with the exception of allowing short‐term rescue medications, such as lorazepam.
6	A history of epilepsy for at least 2 years.
Exclusion	
1	Patient is pregnant or becomes pregnant.
2	A history or condition of progressive brain disorders, unstable systemic diseases, symptomatic cerebrovascular disease, cardiac disease, or alcohol abuse. Special conditions, for example, nonmalignant brain tumors or vascular malformations, can be considered for entry on a case‐by‐case basis at the investigator's discretion.
3	A history or condition of status epilepticus or psychogenic seizures.
4	Presence of a cardiac pacemaker, vagus nerve stimulator, or metal implantation in the body (other than the teeth) including neurostimulators, cochlear implants, and implanted medication pumps.
5	Pervious surgery involving opening of the skull.
6	Allergy to or condition contraindicating lidocaine.
7	Unable to express the presence of pain or discomfort.
8	Allergy to Silver.
9	Participating in other clinical trials.
10	Unable to speak Mandarin in China or English in the US.
11	Unable to knowingly give consent.

### Baseline dense array EEG recordings

Baseline 256‐channel dEEG evaluation of spike frequency was conducted on two separate sessions, with spikes identified by clinician scoring. Each baseline recording was 2 h in duration. Identified spikes were clustered into to spatial similar groups, and each spike cluster was averaged and localized.

### Head model construction

Source localization of each averaged cluster was accomplished with an individual head model constructed from the patient's own MRI, plus an atlas CT that was nonlinearly warped to the MRI for detailed characterization of bone and other tissue conductivities.^14^, ^15^, ^16^ The 256‐electrode sensor positions were co‐registered with the MRI surface precisely in 3‐D space with a geodesic photogrammetry system and verified for accuracy with photographic images. Spike clusters were then localized to the cortical surface.^17^, ^18^

### Target selection

Selection of which spike to target for treatment was based on frequency: the cluster with the highest frequency was selected for stimulation. Spike locations were identified as follows: Patient 1, left lateral temporal (target) and left posterior temporal, left orbitofrontal, left anteromedial temporal, right posterior temporal (nontargets); Patient 2, left anteromedial temporal (target); Patient 3, right anteromedial temporal (target), left anteromedial (nontarget); Patient 4, right anteromedial (target), left anteromedial (nontarget); Patient 5, left anteromedial (target), right posterior temporal (nontarget); Patient 6, left anterior temporal (target), left medial temporal (nontarget); Patient 7, right orbitofrontal‐anterior temporal (target), right medial temporal (nontarget).

Two zones from the target spike were subjected to a “probe” treatment prior to the formal 5‐day treatment protocol. One probe examined the effects of stimulating cortex that corresponded to the temporal onset of the spike, while the second stimulated cortex relating to maximal amplitude of the discharge. The cortical locations of spike onset and amplitude differed in all cases. The probe that resulted in the greatest spike suppression was selected for the 5‐day treatment protocol. In cases where no significant differences were found between onset and maximal amplitude, the cortical location corresponding to spike onset was selected for treatment. The stimulation protocol for each of the two probe sessions consisted of a single 17‐min train of 500 slow (0.5 Hz) cathodal stimulation pulses, with stimulation parameters identical to those during the subsequently administered 5‐day treatment protocol.

### TES treatment protocol

**Figure 1:**
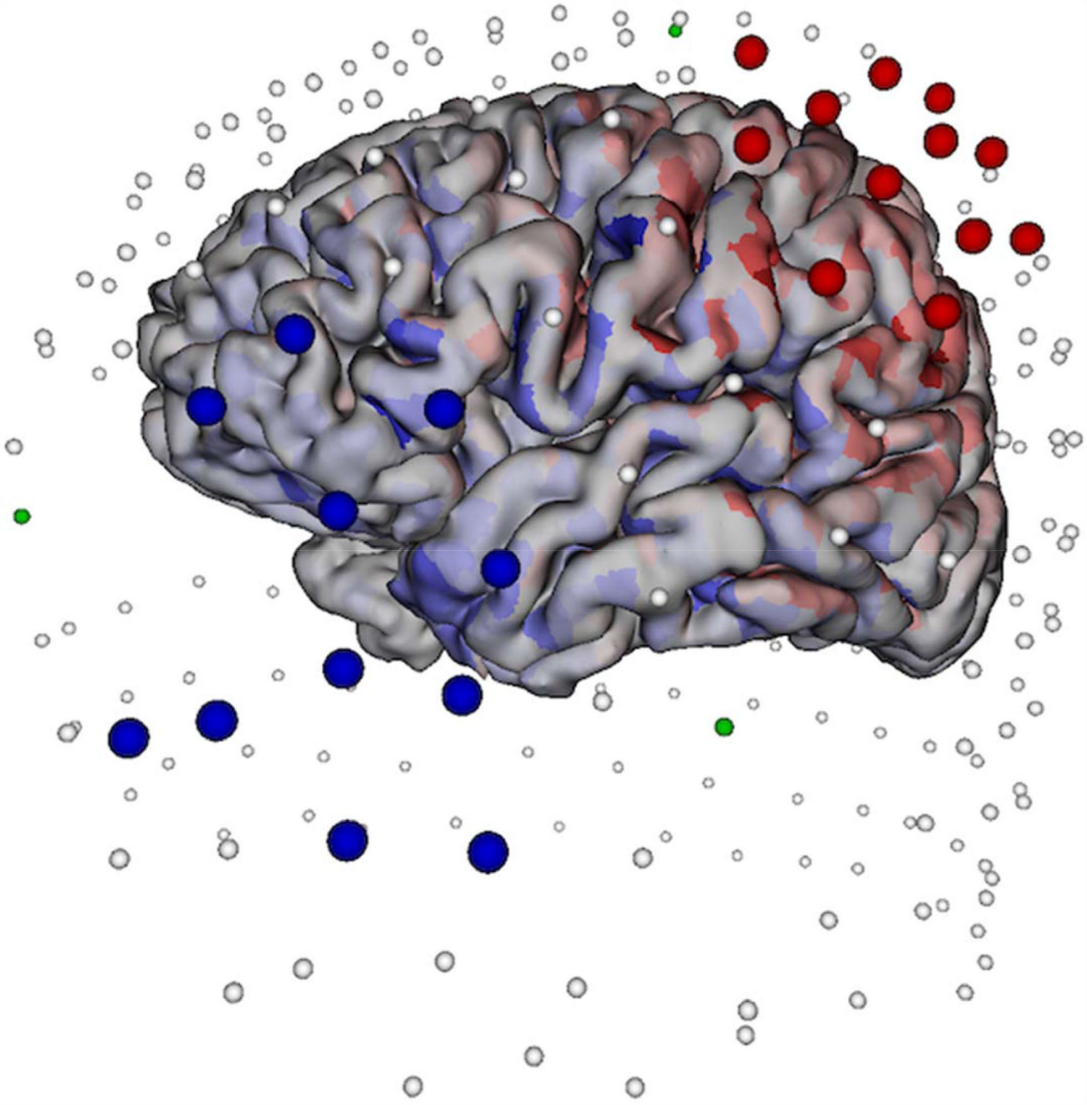
A cloud of source‐sink electrodes selected for TES. The 15 large blue electrodes are those selected as cathodes, and the 15 large red electrodes are those selected as anodes. The arrows pointing away from the left anterior temporal lobe represent the cortical patches selected for target spike cluster localization.

The treatment protocol consisted of five consecutive days of outpatient TES. Each day began with a baseline dEEG recording of at least 20 min and up to an hour in duration. The baseline recording was followed by three 17‐minute trains of 500 cathodal pulses each, with trains separated by a 10‐minute rest interval. Each pulse was 100 ms in duration, with frequency of stimulation set at 0.5 Hz. Total current flow for each pulse did not exceed 2 mA, and current flow for any single electrode did not exceed 200 *u*A. Continuous dEEG recording for 3 h following each of the five TES sessions allowed for the assessment of the posttreatment spike rate, and assured there was no worsening of epileptiform activity before the patient was sent home. The pre‐ and post‐TES dEEG recordings were examined and scored for states of wakefulness, drowsiness, stages 2 and 3–4 NREM sleep, and REM sleep. Spike counts before and after TES sessions were obtained by visual analysis, and spike frequencies (spikes/hour) were calculated before and after each day of TES treatment. Spike counting was conducted with the same procedures both before and after the treatment. The Figure 1 illustrates an array of cathodes (red electrodes) and anodes (blue electrodes) selected from the entire 256‐channel dEEG array for stimulating cortex corresponding to the onset of a left temporal lobe spike.

## Results

The subject's tolerated the treatment sessions well and typically slept through each session. Some subject's experienced mild irritation from the electrode array and three reported occasional phosphenes during the treatment sessions. There was no worsening of the subject's typical epileptiform activity, either clinically or electrographically, during treatment, and no appearance of new epileptiform activity posttreatment for any subject. There were no significant differences in the distribution of waking and sleep states between pre‐ and posttreatment EEG recordings. The pre‐ and posttreatment spike counts were expressed per hour. The spike counts were significantly suppressed acutely (within session) by TES treatment for the targeted spikes for patients 1–5, using a one‐tailed paired‐comparison t‐test (pre vs. post) separately for each subject, with sessions as observations (*N* = 5 sessions for each subject). In total, spikes were suppressed in 29 of 35 treatment days. The nontargeted spikes were also suppressed significantly for four subject's. Spikes were summed for all seven subjects before and after TES for both targeted and nontargeted discharges (Table 2). Spike frequencies did not significantly change for any subject over the course of posttreatment EEG recording. Posttreatment seizures were recorded, but follow‐up data are insufficient for statistical analysis.

**Table 2:** Change in Spike Rate (spikes/hr) from Pretreatment to Posttreatment dEEG.

Target Spike Rate			Nontarget Spike Rate		
Pretreatment	Posttreatment	% Change	Pretreatment	Posttreatment	% Change
Patient 1					
129	79	−39%	57	22	−61%
82	21	−74%	14	4	−71%
137	68	−50%	82	17	−79%
187	84	−55%	76	12	−84%
214	132	−38%	60	20	−67%
Patient 2					
89	57	−36%	–	–	–
40	36	−10%	–	–	–
141	80	−43%	–	–	–
36	4	−89%	–	–	–
34	12	−65%	–	–	–
Patient 3					
42	18	−57%	16	3	−81%
55	32	−42%	10	8	−20%
46	33	−28%	25	4	−84%
66	18	−73%	33	9	−73%
30	33	10%	12	8	−33%
Patient 4					
23	25	9%	38	29	−24%
85	22	−74%	3	4	33%
104	53	−49%	11	13	18%
44	16	−64%	6	8	33%
74	13	−82%	0	23	nv
Patient 5					
84	35	−58%	39	9	−77%
44	31	−29%	9	18	91%
32	16	−50%	18	2	−89%
14	16	12%	29	7	−75%
17	13	−25%	26	5	−80%
Patient 6					
278	96	−65%	41	44	7%
33	38	15%	98	26	−73%
128	94	−27%	196	76	−61%
68	286	321%	1168	1527	31%
848	380	−55%	623	317	−49%
Patient 7					
23	2	−91%	37	5	−86%
108	38	−63%	0	0	‐‐
44	56	27%	16	3	−81%
327	43	−87%	44	1	−98%
32	2	−94%	65	8	−88%
Mean % change (all subjects)		−35%	Mean % change (all subjects)		−38%

Statistical significance	Target	Nontarget
Patient 1	*P* < 0.001	*P* < 0.01
Patient 2	*P* < 0.02	–
Patient 3	*P* < 0.04	*P* < 0.03
Patient 4	*P* < 0.02	*P* > 0.3 (ns)
Patient 5	*P* < 0.07	*P* < 0.04
Patient 6	*P* > 0.3 (ns)	*P* > 0.4 (ns)
Patient 7	*P* > 0.1 (ns)	*P* < 0.03

Spike Sums	Target		Nontarget	
	Pre	Post	Pre	Post
Patient 1	749	384	289	75
Patient 2	340	189	–	–
Patient 3	239	134	96	32
Patient 4	330	129	58	77
Patient 5	191	111	120	41
Patient 6	1355	894	2126	1990
Patient 7	529	141	162	17

## Discussion

Monitoring of dEEG during and after treatment affirmed the safety of the TES protocol in each patient. There was no evidence of seizure discharges, no evidence of after‐discharges induced by the TES pulses, and no evidence of worsening of spike rate.

Targeted spikes were suppressed by TES treatment as assessed by statistical analysis of each patient's pretreatment versus posttreatment spike rates over the 5 days of treatment in five of seven patients, and in all five treatment sessions in two subjects. These preliminary results are consistent with the hypothesis that noninvasive electrical neuromodulation is safe when used within safety guidelines, and may be effective in suppressing cortical excitability when used with the appropriate protocol.^13^. Our results may be a harbinger of novel techniques that can be developed for long‐term management of difficult epilepsy.

Nontarget spikes, likely projected in some cases, were also suppressed in four of six patients, even though the TES targeting was directed at the opposite temporal lobe in two cases. Nontarget spikes were not increased in any subject. Epilepsy is a network problem, and both targeted and nontargeted spikes reflect components of that network. We speculate that suppressing spikes in any part of the epileptic network affects the whole to some degree, regardless of the specific location that is primarily targeted. We do not believe that TES targeting of random brain region produces equivalent results. Rather, only focal targeting of a node within an epileptic network is likely to be effective in spike suppression, supporting the rationale that treatments utilize realistic patient‐specific head models in conjunction with high‐density EEG recordings to optimize spatial resolution. Future research will be necessary to confirm the superiority of specific targeting compared to random targeting, or techniques using standard EEG or large electrodes directed to the general location of the seizure focus.

Spike suppression does not imply seizure suppression. It will be important in future studies to include a placebo treatment control and to document seizure, as well as spike, suppression. Future research in TES for epilepsy may include changing treatment parameters, including increasing duration of treatment, safely increasing the amount of current that can be delivered to selected targets, repeated treatment sessions over time, and employing newer technologies that deliver more precise and focused current to deep brain structures via temporally interfering electrical fields.^19^ We also predict that advances will occur with greater understanding of patient‐specific epileptic networks.^20^

## Author Contributions

Conception and design of the study: MDH, Pl, DMT. Acquisition and Data Analysis: MDH, RF, MW, CM, CR, JW, PL, JH, LP, DMT**. **Drafting Manuscript and Figure Preparation: MDH, MW, PL, DMT.

## Conflicts of Interest

JH, PL, and DMT were employees of EGI (now Philips Neuro) during the time that this research was conducted. No authors are current employees of Philips Neuro. None of the authors have any conflict of interest, financial, or otherwise.
